# Selective vulnerability of motor neuron types and functional groups to degeneration in amyotrophic lateral sclerosis: review of the neurobiological mechanisms and functional correlates

**DOI:** 10.1007/s00429-023-02728-6

**Published:** 2023-11-24

**Authors:** Saak V. Ovsepian, Valerie B. O’Leary, Salvador Martinez

**Affiliations:** 1 Faculty of Engineering and Science, University of Greenwich London, Chatham Maritime, Kent, ME4 4TB UK; 2 Department of Medical Genetics, Third Faculty of Medicine, Charles University, Ruská 87, 10000 Prague, Czech Republic; 3 Instituto de Neurociencias UMH-CSIC, Avda. Ramon y Cajal, 03550 San Juan de Alicante, Spain; 4 Center of Biomedical Network Research on Mental Health (CIBERSAM), ISCIII, Madrid, Spain

**Keywords:** Motor neuron disease, SOD1 mutation, TDP43, Bulbar and spinal ALS, Motor units, Skeletal and visceral muscles, Excitotoxicity

## Abstract

Amyotrophic lateral sclerosis (ALS) is a fatal neurodegenerative condition characterised by a progressive loss of motor neurons controlling voluntary muscle activity. The disease manifests through a variety of motor dysfunctions related to the extent of damage and loss of neurons at different anatomical locations. Despite extensive research, it remains unclear why some motor neurons are especially susceptible to the disease, while others are affected less or even spared. In this article, we review the neurobiological mechanisms, neurochemical profiles, and morpho-functional characteristics of various motor neuron groups and types of motor units implicated in their differential exposure to degeneration. We discuss specific cell-autonomous (intrinsic) and extrinsic factors influencing the vulnerability gradient of motor units and motor neuron types to ALS, with their impact on disease manifestation, course, and prognosis, as revealed in preclinical and clinical studies. We consider the outstanding challenges and emerging opportunities for interpreting the phenotypic and mechanistic variability of the disease to identify targets for clinical interventions.

## ALS spectrum

Motor neurons (MNs) are a specialised cell type of the central nervous system (CNS) supporting motor functions through coordinated activation of skeletal and visceral muscles. Accordingly, functional deficit in MNs due to intoxication, trauma, stroke, or degeneration leads to weakness and atrophy of muscles with motor impairments. Due to their control of vital processes and functions, injury and loss of MNs can lead to life-threatening muscular paralysis, with respiratory failure marking the end point of lethal cases. As a unique cell type, MNs can be affected by a specific group of neurodegenerative conditions (Goutman et al. 2022). This family of rare diseases includes spinal muscular atrophy (SMA), progressive bulbar palsy (PBL), primary lateral sclerosis (PLS), and amyotrophic lateral sclerosis (ALS), which are the most prevalent degenerative disease of MNs. Affecting 2–3 persons per 100,000, over ~ 90% of ALS cases are idiopathic with no identifiable cause, while the remaining ~ 10% run in families and are associated with genetic aberrations (Kiernan et al. 2011; Goutman et al. 2022).

While generally considered a disease of MNs, there is growing recognition of ALS as a multisystem disorder (Swinnen and Robberecht 2014; Grossman 2019). The composite nature of the disease is supported not only by the fact that a significant proportion of ALS patients develop a frontotemporal, extrapyramidal, cerebellar as well as multiple visceral and non-motor dysfunctions (Swinnen and Robberecht 2014; Strong et al. 2017; Masrori and Van Damme 2020), but also by variations of the disease amongst distinct cultural, geographical, and genetic groups (Swinnen and Robberecht 2014; Gajdusek and Salazar 1982; Plato et al. 2003; Chio et al. 2013). Indeed, after age and sex adjustments, ALS incidents amongst African, Asian, and Hispanic ethnicity are significantly lower than in non-Hispanic Caucasians (Alappat 2007; Marin et al. 2017; Collaborators 2018). A comparison of ALS incidents in European countries shows the highest rates in Finland, while Italy has the lowest (Collaborators 2018; Alappat 2007; Marin et al. 2017). Mounting data also suggest significant effects of gender, occupation, and physical exercise on the prevalence, trajectory, and severity of ALS (Julian et al. 2021; McCombe and Henderson 2010). Finally, environmental, and genetic factors appear to influence the progression and prognosis of the disease, with racially heterogeneous or admixed populations with ALS living longer than homogeneous White or Black (Collaborators 2018; Kiernan et al. 2011).

Most cases of ALS set on with muscle weakness and spasticity in the limbs, indicating a predominantly spinal form of the disease, which shows a survival median of 3–5 years, while in ~ 20% of cases, the onset is bulbar, leading to speech and swallowing problems with the worst prognosis, yielding an average survival of ~ 2 years (Swinnen and Robberecht 2014; Kiernan et al. 2011). The causes and mechanisms of the differences in the prevalence of ALS in different groups and the site of the onset and severity of motor phenotype remain unclear, with multiple players emerging in the contribution (Ingre et al. 2015; Su et al. 2021; Yang et al. 2022; Kiernan et al. 2021; Ragagnin et al. 2019). As detailed in the following, multiple characteristics of MNs, including their intrinsic excitability, synaptic connections, and chemical microenvironment, seem to be at play, which not only provide clues for the phenotypic variability of ALS and elucidate the multisystem characteristics of the disease, but may also facilitate translational research and potential therapeutic interventions.

## Heterogeneity of MNs and their vulnerability gradient to ALS

MNs is an umbrella term describing a heterogeneous population of neurons which supports and coordinates a wide range of somatic and visceral motor activities throughout the entire body axis. In addition to the general division of MNs into cerebral (upper) and bulbospinal (lower), there are remarkable morphological and physiological differences between MNs organised in various functional groups (Stifani 2014; Ragagnin et al. 2019; Fitzpatrick 2001; Baczyk et al. 2022a). Specifically, the lower MNs of the brain stem and spinal cord subdivide into three groups: (1) somatic, (2) special visceral, and (3) general visceral (Fitzpatrick 2001). With soma in the anterior horn of the spinal cord and motor nuclei of III, IV, V, VI, VII, IX, X and XII cranial nerves in the brainstem, somatic MNs are specialised further into α-, β- and γ-MN types (Fitzpatrick 2001; Stifani 2014; Baczyk et al. 2022a). The large α-MNs control the bulk of force-generating elements of skeletal muscles of the body (Stifani 2014). Working as functional assemblies, they drive and coordinate the voluntary contraction of specific muscle groups and control the general muscular tone and activity. Two other MN types, which control the tonus and strength of muscle contraction, are (1) β-MNs innervating intrafusal and extrafusal fibres of muscle spindles, and (2) γ-MNs responsible for tuning the sensitivity of muscle stretching via innervation of intrafusal spindles (Fitzpatrick 2001; Scott et al. 2001; Burke et al. 1971). Special and general visceral MNs, on the other hand, are responsible for innervation of specialised muscle groups, with the former controlling the facial expression, mastication, swallowing, and phonation through cranial nerves, and the latter regulating the tonus of the cardiac and smooth muscles of the visceral organs, through synapsing on neurons of autonomic ganglia, which in turn controls the muscle activity of the inner organs (Fitzpatrick 2001; Scott et al. 2001).

**Fig. 1:**
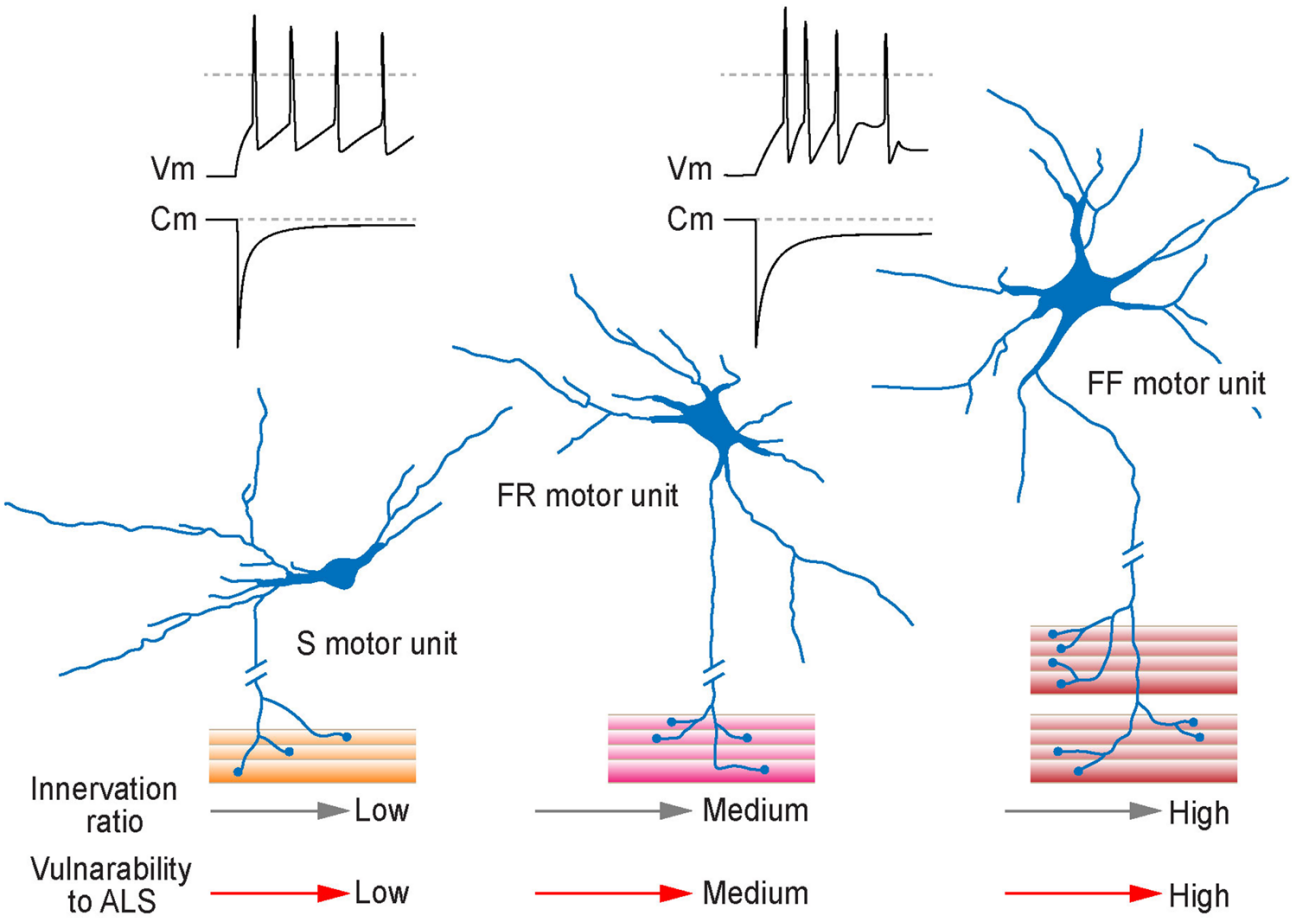
Schematic drawings of the three main types of motor units with the relationship of MN size, muscle fibre innervation ratio and vulnerability to ALS (grey and red arrows, respectively). From left to right: S, FR and FF (slow, fatigue-resistant and fast-fatiguable, respectively) motor units, with increasing number of innervated muscle fibres per MN and a general trend of increasing MN size. Differences in MN size impact their membrane capacitance (*C*_m_) and excitability-firing activity (*V*_m_) in response to depolarising inputs (black traces of membrane capacitance and excitability-firing activity of small and large MNs of the trigeminal motor nucleus). Differences in colouring of muscle fibres designate specifics of the energetic requirements and degree of reliance of muscle fibres on ATP produced by anaerobic or aerobic synthesis as source of energy for contraction (left to right—from oxidative phosphorylation to glycolysis)

The structural and functional differences of various MN types reflect their evolutionary specialisation as part of motor units supporting different functions (Mendell 2005; Henneman et al. 1965; Button et al. 2007; Gardiner 1993) **(**Fig. 1**)**. As a critical constituent of motor activity, the motor unit comprises MN with a group of muscle fibres innervated by its axon via specialised connections known as neuromuscular junctions (NMJ). Based on the strength–contraction characteristics and energetic requirements of the muscle fibres, motor units organise into fast-twitch fatiguable (FF), fast-twitch fatigue-resistant (FR) and fatigue-resistant slow-twitch (S) types (Burke et al. 1971; Ragagnin et al. 2019; Burke 1999). The activity of FF muscle fibres relies largely on glycolytic (anaerobic) energy production and is the primary generator of strong muscular contractions controlled by fast-firing α-MNs, which have larger sizes and thick myelinated axons. These units typically contain over 300–500 muscle fibres, with their number in big muscles of the trunk and limbs reaching up to 2000 (Burke et al. 1974, 1971; Burke and Tsairis 1973). Distinctly, the activity of motor units with FR fibres relies on the supply of combined glycolytic and oxidative (aerobic) energy, involving fewer muscle fibres and generating weaker, fatigue-resistant contraction (Highstein et al. 1982; Picard et al. 2012). The third motor unit type is formed primarily of 200 or fewer S-type fibres generating weak but sustained contraction, with fibre activity largely reliant on energy supply by oxidative metabolism, capable of maintaining muscle contraction for an hour and longer (Burke 1999; Picard et al. 2012). The specialisation of MNs to drive the activity of muscle fibres with different characteristics and functional role appears to be of prime relevance to their vulnerability to ALS (Comley et al. 2015, 2016; Nijssen et al. 2017; Pun et al. 2006). Although multiple factors are at play in setting the gradient of MN sensitivity to neurodegeneration, they are tentatively divided into neuronal (intrinsic and extrinsic) and non-neuronal, with the latter mediated largely by paracrine effects of neuromodulators and trophic factors released from muscle fibres, supporting glia and immune cells.

## Intrinsic factors influencing MN vulnerability to neurodegeneration of ALS

MNs of different motor units show distinct activity characteristics influenced by their intrinsic properties, such as the size and morphology, consortium of ion channels and transporters with their regulation, and mechanisms governing intracellular Ca^2+^ dynamics (Button et al. 2007; Gardiner 1993; Henneman et al. 1965; Highstein et al. 1982; Manuel and Zytnicki 2019; Russo and Hounsgaard 1999; Ruegsegger et al. 2016; Baczyk et al. 2022b). The differences in the size of dendritic tree set the electrical capacitance of MNs and rheobase, while the collection of ion channels supporting firing activity and shaping post-discharge recovery defines kinetics, frequency and duration of their output to muscle fibres (Henneman et al. 1965; Gardiner 1993; Baczyk et al. 2022b). In general, MNs of S units with higher intrinsic excitability and compact dendritic trees respond more readily to depolarising inputs (Burke et al. 1982; Cullheim et al. 1987; Kernell and Zwaagstra 1981). Autopsy reports of ALS patients show that surviving MNs of the spinal cord have, on average, smaller soma and possibly higher intrinsic excitability (Kawamura et al. 1981; Dengler et al. 1990). Accordingly, more excitable and in average smaller MNs of oculomotor, trochlear and abducens nuclei innervating ocular muscles are largely spared in ALS until the end stage of the disease (Gizzi et al. 1992; Okamoto et al. 1993), permitting eye-tracking devices as communication tools for fully paralysed patients (Caligari et al. 2013). Likewise, MNs of Onuf’s nucleus innervating striatal muscles of the pelvic bed with lower rheobase and higher intrinsic excitability (Hochman et al. 1991; Sasaki 1991) remain relatively intact in advanced ALS patients (Iwata and Hirano 1978; Mannen et al. 1977, 1982). While these findings are in line with greater sensitivity of large spinal cord MNs to ALS and better survival of MNs of small motor units, the effects of disease-related neuronal shrinkage and counting bias introduced by ALS-resistant smaller intrafusal MNs in autopsy samples should be cautiously considered.

**Fig. 2:**
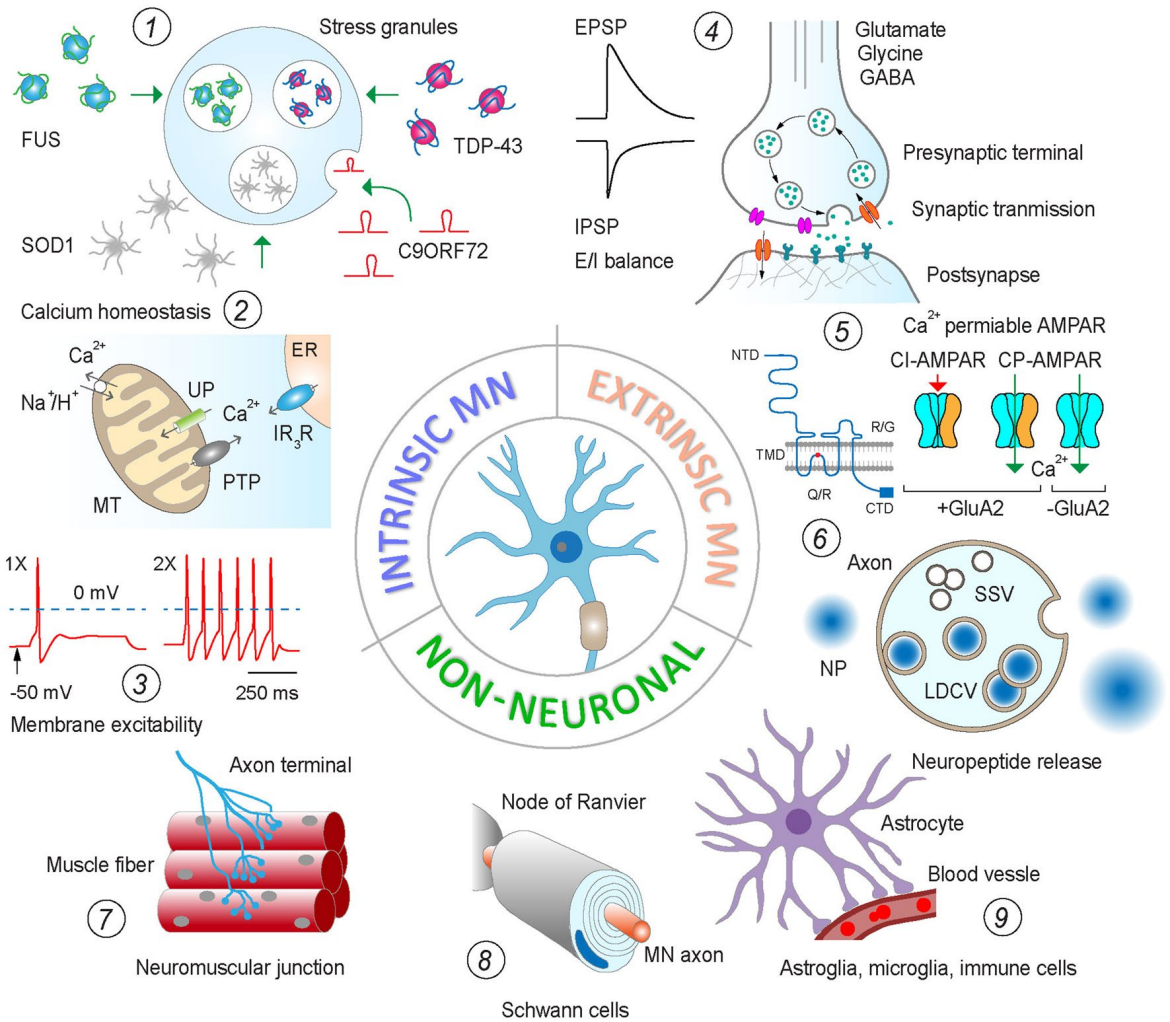
Summary diagram illustrating the principal intrinsic (cell autonomous, MNs), extrinsic neuronal and non-neuronal factors influencing the vulnerability of MNs to ALS. Among intrinsic MN factors, (1) cellular stress response to RNA and protein granules, (2) disruption of mechanisms regulating Ca^2+^ dynamics and (3) changes in membrane excitability are most widely discussed. The extrinsic influence on MN vulnerability to ALS, on the other hand, is mediated mainly via (4) a shift of E/I balance at MN inputs, (5) higher expression of Ca^2+^-permeable GluA2 subunit in AMPA receptors and (6) paracrine effects of neuropeptides and hormones. Finally, there is rising evidence for the role of trophic factors released by (7) muscle fibres at NMJ, (8) peripheral glia (Schwann cells and satellite glia) and (9) astrocytes and immune cells in the central nervous system. Specific factors and mechanisms mediating synaptic and paracrine effects are discussed throughout the review. *FUS* fused in sarcoma RAN-binding protein, *SOD1* superoxide dismutase, *TDP-43* transactive response DNA-binding protein, *C9ORF72* C9 open reading frame 72 protein, *MT* mitochondria, *PTP* permeability transition pore, *UP* uniporter, *ER* endoplasmic reticulum, *EPSP* excitatory postsynaptic potential, *IPSP* inhibitory postsynaptic potential, *CI* calcium impermeable, *NTD (CTD)* N- and C-terminal domain, *TMD* transmembrane domain, *GluA2* subunit of AMPA receptor, *NP* neuropeptide, *SSV* small synaptic vesicle, *LDCV* large dense core vesicle

With regard to MN excitability and resistance to ALS degeneration, mechanisms controlling intracellular Ca^2+^ dynamics by endoplasmic reticulum (ER) and mitochondria, and differential expression of Ca^2+^ binding proteins are of particular interest (Grosskreutz et al. 2010; Leal and Gomes 2015; Verma et al. 2022a; Wainger et al. 2014) **(**Fig. 2**)**. With reported higher levels of calbindin D28k and parvalbumin in ALS-resistant MNs (Alexianu et al. 1994; Comley et al. 2015), tight regulation of intracellular Ca^2+^ dynamics is considered protective for MNs (Grosskreutz et al. 2010; Verma et al. 2022a). In this context, the differential expression of ER chaperones calreticulin and SIL1 has raised considerable interest (Bernard-Marissal et al. 2012, 2015; Filezac de L'Etang et al. 2015; Ragagnin et al. 2019). While the former is a multifunctional Ca^2+^-binding protein, regulating its signalling activity and controlling the export of proteins from ER, the latter tunes the ER response to stressors and related secretory mechanisms. With both expressed in high amounts in ALS-resistant MNs, their level gradually declines with disease progression in preclinical models, while targeted overexpression of Ca^2+^ binding proteins in ALS MNs extended their survival and increased the life span of experimental mice (Nijssen et al. 2017). Longitudinal analysis and comparison of three SOD1 models (G93A, G85R and hSOD1) with different developmental onset and progression of motor deficit and MN degeneration showed a positive correlation of the ER stress response with the disease manifestation and loss of MNs (Saxena et al. 2009). MNs with higher vulnerability to degeneration exhibited stronger ER stress response judged on the expression of stress markers from early stages of development. While in ALS, both vulnerable and resistant MNs showed a developmental increase in ubiquitin signals, those with higher susceptibility to degeneration showed stronger stress response associated with microglia activation (Baczyk et al. 2022b; Saxena et al. 2009). Saxena and co-workers suggested a mechanistic relationship between the excitability and intrinsic neuroprotection mechanisms of FF motor units, with enhancing MN activity triggering neuroprotective response of MNs in SOD1 mice, while reducing excitability accelerating neurodegeneration and motor decline (Saxena et al. 2013). This mechanism seems to be negatively regulated by metabotropic cholinergic drive through ER stress response and mTOR activation (Saxena et al. 2013; Baczyk et al. 2022b).

Another intrinsic factor considered in the context of the selective vulnerability of MNs to ALS is matrix metalloprotease 9 (MMP9) (Kaplan et al. 2014; Spiller et al. 2019). This Zn^2+^-activated protease is functionally related to various cell surface proteins, including integrins, TrkA and TrkB receptors, and plays essential roles in neurodevelopment and synaptic plasticity (Verslegers et al. 2013; Vafadari et al. 2016) and regulation of axonal myelination and pruning (Reinhard et al. 2015). MMP9 polymorphisms are among the principal risk factors for sporadic ALS (Zawislak et al. 2009; Pabian-Jewula and Rylski 2023), with results of postmortem studies of ALS patients showing MMP9 levels ~ 2.5-fold lower in the oculomotor nucleus as compared to lumbar MNs (Brockington et al. 2013). Accordingly, Kaplan and co-workers reported higher expression of MMP9 in ALS-vulnerable MNs of the SOD1 mouse model, with its overexpression in wild-type MNs sufficient to trigger axonal dieback (Kaplan et al. 2014). Likewise, targeted expression of osteopontin in α-MNs, which is known to enhance MMP9 production through activation of the αv33 integrin receptor, promoted the degeneration of FR/S MNs in the SOD1 mouse model (Morisaki et al. 2016). In agreement with these findings, lowering MMP9 levels or its genetic removal was shown to extend the survival of MNs (Kaplan et al. 2014; Kiaei et al. 2007) with protective effects in SOD1 and TDP-43 ALS mice (Lorenzl et al. 2006; Kaplan et al. 2014; Spiller et al. 2019).

## Extrinsic neuronal factors influencing MN vulnerability to neurodegeneration of ALS

In addition to differences in the intrinsic properties, various functional groups of MNs are under differential influence by their synaptic inputs and neurochemical microenvironment. Synaptic and non-synaptic membrane currents not only regulate the development and integration of MN in functional networks, but also play an essential role in their survival and plasticity (Kalb and Hockfield 1992; Stegenga and Kalb 2001; Inglis et al. 2000; Ovsepian and Vesselkin 2006, 2014). Similar to the described intrinsic effects, synaptic activity in MNs influences their vulnerability to ALS (Nijssen et al. 2017). Several groups have demonstrated that changes in the balance of excitatory/inhibitory (E/I) drives are of crucial importance to neurodegeneration in ALS, with the E/I ratio highest in FF MNs—the most vulnerable neuron type (Nijssen et al. 2017; Kanai et al. 2012). Increase in excitatory synaptic drive as a contributor to ALS is supported by overall loss of excitatory amino acid transporter 2 (EAAT2) in the motor cortex and spinal cord of ALS patients, which could lead to glutamate excitotoxicity (Rothstein et al. 1995). The balance of E/I synaptic inputs and parameters of the excitatory drive are essential regulators of Ca^2+^ dynamics in MNs and other neuron types, which, if disrupted, could lead to impairments of signalling, causing abnormal activity of neurons with cytotoxicity and cell death (Van Den Bosch et al. 2006; Leal and Gomes 2015; Ovsepian and Friel 2008). The inherently lower Ca^2+^ buffering of ALS-sensitive MNs is aggravated by high expression of Ca^2+^-permeable AMPA receptors (AMPAR) comprising the unedited glutamate receptor 2 (GluR2) subunit (Grosskreutz et al. 2010; Van Den Bosch et al. 2006; Greig et al. 2000; Zanganeh et al. 2022) **(**Fig. 2**).** With over 1/3 of AMPAR being permeable for Ca^2+^, the larger Ca^2+^ influx strains the ER and mitochondrial calcium buffering of MNs and increases the risks of cell stress and degeneration (Greig et al. 2000). Notably, the level of edited GluR2 transcripts in ALS-resistant oculomotor neurons of brain autopsies is significantly higher (Brockington et al. 2013). Although the total transcript level of GluR2 in the spinal cord of ALS patients is comparable to that in healthy, post-translational editing fails in almost half of all transcripts, leading to an increase in the level of Ca^2+^-permeable AMPAR with enhanced AMPAR-mediated Ca^2+^ currents (Kwak and Kawahara 2005). In support of the contribution of unedited GluR2 to the vulnerability gradient of MNs to ALS, the Ca^2+^ current mediated by AMPAR of oculomotor MNs is smaller as compared to that in large spinal cord MNs (Brockington et al. 2013). A recent analysis showed that experimental manipulations of the expression of GluR2 gene and RNA could tune the sensitivity of MNs in the SOD1 mouse model of ALS (Zanganeh et al. 2022).

The importance of E/I balance in influencing the vulnerability gradient for MNs in ALS is also showcased by studies of inhibitory synaptic inputs. ALS patients display cortical hyperexcitability that is thought to arise from E/I imbalance in MNs therein. The underlying mechanisms of this change remain unclear, but ALS patients appear to have lower expression of GABA_A_ receptors (GABA_A_R) in the motor cortex and a generally lower efficacy of inhibitory inputs (Petri et al. 2003; Nieto-Gonzalez et al. 2011). In FF MNs of rat spinal cord, inhibitory postsynaptic currents are predominantly glycinergic (Lorenzo et al. 2006), whereas in oculomotor MNs of the same model, the inhibitory synaptic drive is mediated mainly by GABA_A_R (Brockington et al. 2013; Comley et al. 2015; Lorenzo et al. 2006). Congruously, in ALS patients, the expression of GABA_A_R α-subunit is significantly higher in oculomotor MNs as compared to those of the spinal cord and remains unchanged until the end stage of the disease, suggesting its contribution to the greater resilience of the former to neurodegeneration (Hedlund et al. 2010; Comley et al. 2015). Detailed analysis with comparison of the subunit composition, expression pattern, density, and localisation of inhibitory receptors in ALS-sensitive (trigeminal, facial and hypoglossal) and ALS-resistant (oculomotor, trochlear and abducens) MNs of cranial nerves in rats revealed a significant correlation between the subunit composition of GABA_A_R, glycine/GABA_A_R ratios and frequency of extra-synaptic versus synaptic GABA_A_R with the degree of vulnerability of MNs to degeneration (Lorenzo et al. 2006; Nijssen et al. 2017). These differences contrast the relatively uniform density of gephyrin cluster and synaptic glycinergic receptors levels measured in the same motor nuclei (Lorenzo et al. 2006), with GlyR reported to be significantly reduced in the spinal cord of ALS patients (Hayashi et al. 1981). In this context, it is important to note that ALS-resistant MNs of Onuf’s nucleus do not receive recurrent inhibitory inputs, which in ALS-sensitive MNs are glycinergic (Sasaki 1994; Schellino et al. 2020).

In addition to the E/I ratio, growing data supports the non-autonomous effects of neuropeptides and neurotrophic factors on the vulnerability of MNs to ALS. This premise has been supported by reports demonstrating ALS-resistant MNs in Onuf’s nucleus receiving dense innervations by enkephalin-, somatostatin-, CGRP-, neuropeptide Y- and neurokinin-positive terminals, as well as thyrotropin-releasing hormone-positive axons (Schellino et al. 2020; Gibson et al. 1988; Katagiri et al. 1988; Pullen et al. 1997). The strong neuropeptide and neurotrophic drives have been suggested to render MNs of Onuf’s nucleus comparable to neurons of the autonomic nervous system, which also have a higher resistance to ALS (Schellino et al. 2020). Notably, the protective effect of neuropeptides on Onuf’s MNs is ALS specific, as they succumb to neurodegenerative conditions affecting parasympathetic neurons, such as Fabry’s disease, multiple systems atrophy, Shy–Drager syndrome and Parkinson's disease (Schellino et al. 2020).

## Non-neuronal effects influencing MN vulnerability to neurodegeneration of ALS

Unlike any other neuron type in CNS, MNs extend their axons to reach target muscles at the periphery, forming highly specialised synaptic connections beyond the defence line of the blood–brain barriers. This unique arrangement not only makes MNs subject to retrograde influence by an array of trophic factors and messengers produced at the periphery by muscle fibres, Schwann and satellite cells, but also exposes them to a range of pathogens, toxins and environmental factors (Henderson et al. 1993; Ovsepian et al. 2019, 2016; Cain et al. 2019; Sleigh et al. 2019). Considerable data suggests NMJ as the site of the onset of MN degeneration (Dadon-Nachum et al. 2011; Tsitkanou et al. 2019), with the mediators and mechanisms of peripheral effects on MN vulnerability to ALS remaining elusive.

Evidence from preclinical models and developmental studies indicate protective and neuroplastic effects of several trophic factors and metabolites released by muscles (Tovar et al. 2014; Saini et al. 2021; Taylor et al. 2007) **(**Fig. 2**)**. Multiple reports showed an inverse correlation between the propensity of the axon terminal sprouting and plasticity in muscle with the vulnerability of source MNs to ALS (Frey et al. 2000; Seijffers et al. 2014; Verma et al. 2022b; Pun et al. 2006). A pioneering work by Pun and collaborators showed that in SOD1^G93A^ and SOD1^G85R^ ALS models, axons of FF MNs deteriorate long before the onset of motor symptoms, while axons of FR MNs break down with the onset of motor deficit (Pun et al. 2006). The impairment of MN axons leads to stalling synaptic vesicles at the presynaptic terminal of NMJ with a buildup of apoptotic marker BC12a1-a, which can be alleviated by ciliary neurotrophic factor. The authors conclude that the vulnerability of MNs to ALS is selective and predictable and might be related to the mechanisms of remodelling of presynaptic terminals at NMJ (Pun et al. 2006). In support of these findings, the overexpression of the activating transcription factor 3 (ATF3) in muscles of ALS mice stimulated the sprouting of axon terminals and delayed the onset of MN neurodegeneration (Seijffers et al. 2014). Overexpression of ephrin receptor A4 (EPHA4) in MNs, on the other hand, impairs the reinnervation of NMJs (Van Hoecke et al. 2012) with EPHA4 level changes in the blood of patients correlating with the onset of clinical ALS (Van Hoecke et al. 2012). In SOD1 mice, haploinsufficiency of EprA4 extended MN survival and increased NMJ formation, whereas, in TDP43 mice, it rescued axonopathy (Van Hoecke et al. 2012). Surprisingly, overexpression of peroxisome proliferator-activated receptor gamma coactive 1-alpha (PGC-1α), known to regulate muscle fibre types and their response to metabolic demands while increasing the oxidative capacity of myotubes and fatigue resistance, did not affect the onset of ALS signs or alter the survival of transgenic mice (Da Cruz et al. 2012). It is interesting to note that the level of neuromuscular activity with activity-dependent reprograming of FF motor units to less forceful, slower FR types could reduce their vulnerability to ALS (Gordon et al. 2010). In a partially denervated hind limb muscle of SOD1 mice, the rapid age-dependent decline in fast twitching muscles associates with significantly higher number of type IIA and type IID/X fibres attributed to FR slower units with higher resistance to ALS (Gordon et al. 2010).

Studies of paracrine effects of glial cells on MN survival in ALS suggested their likely influence (Philips and Rothstein 2014; Valori et al. 2014; Takahashi 2023) **(**Fig. 2**)**. The removal of astrocytes in SOD1 mice, for instance, increased their survival without effects on the disease onset, while overexpression of SOD1^G93A^ in astrocytes has toxic effects on MNs (Yamanaka et al. 2008; Yamanaka and Komine 2018). The relevance of these mechanisms to the vulnerability of various MN groups of ALS patients remains to be shown. Likewise, the depletion of mutant SOD1 from microglia resulted in an extended life span of mice with slower disease progression (Boillee et al. 2006; Christoforidou et al. 2020). The mechanisms underlying the protective effects of microglia in ALS remain to be elucidated. Interestingly, there have been no reports of the influence of oligodendroglia and Schwann cells on the endurance of ALS MNs of humans, even though during development, these cells play a crucial role in MN differentiation and functional integration (Lee et al. 2012; Horner et al. 2021). In animal models, unlike astrocytes, microglia, and oligodendrocytes, where suppression of mutated SOD1^G37R^ can confer protection on MNs, downregulation of this protein in Schwann cells accelerated the progression in ALS phenotype without effects on the onset of motor impairments (Lobsiger et al. 2009). This response could be partly due to attenuation in IGF-1 effects in the MN axons, leading to the reduction of the capacity of MNs to regenerate and form new contacts with muscle fibres. It is noteworthy that Schwann cells of axon terminals innervating different populations of muscle fibre show different levels of axon-repellent Semaphorin 3A, with postnatal, regenerative and paralysis-induced remodelling of neuromuscular connection accompanied by increased expression of this protein on FF muscle fibres (De Winter et al. 2006). Based on the semaphorin 3A expression analysis by terminal Schwann cells in the SOD1 mouse model, the authors suggest that it suppresses terminal sprouting under stress and contributes to their early and selective loss in ALS (De Winter et al. 2006). On the same note, grafts of bone marrow mononuclear cells in the muscles of SOD1 ALS and muscle-deficient mouse models yielded GDNF-dependent protective effects, manifested in better NMJ stability and extended MN survival (Pastor et al. 2013; Rando et al. 2018). In the context of long-range retrograde effects of muscles on the viability of motor units, it is worth noting that MNs of Onuf’s nucleus are under the protective influence of testosterone of target bulbospongiosus muscles, with its deficiency leading to the death of MNs (Schellino et al. 2020; Little et al. 2009). These findings agree with the results of an earlier study showing that castration can cause retardation of Onuf’s MNs and a reduction in size (Vercelli and Cracco 1990).

## Implications of differential MN vulnerability to ALS for clinical translation

In addition to gaining mechanistic insights into the pathobiology of ALS, research into the differential vulnerability of motor units has important implications for unveiling therapeutic targets and avenues for the protection and rescue of motor functions. Because of the limited access to degenerating MN in the human nervous system, most concepts and strategies used in translational studies of ALS have relied on data from animal models of familial variants of ALS (Mead et al. 2023; Perrin 2014). With their numerous advantages in recapitulating specific features of familial ALS, genetic models fall short of capturing the complexity of familial and sporadic variants, with the latter comprising over 90% of ALS cases (Todd and Petrucelli 2022). For instance, cytoplasmic aggregations of TDP-43 DNA/RNA binding protein observed in TDP-43 mice, which represent the hallmark of degenerating human MNs, are not observed in SOD1 and FUS (fused in sarcoma) mouse models (Vance et al. 2009; Mackenzie et al. 2007). Another limitation of the TDP-43 model is that under physiological conditions, its level is tightly autoregulated, while in transgenic models, the expression of TDP-43 is pathologically enhanced (Tsao et al. 2012). Therefore, the TDP-43 toxicity in preclinical models may not fully capture the features of ALS proteinopathy with effects on MNs (de Boer et al. 2020; Tsao et al. 2012). Histopathological analysis of the most common genetic subtype of ALS linked with expansion of GGGGCC hexanucleotide in the *C9orf72* gene, in addition to cytoplasmic TDP-43 aggregates, shows p62-positive deposits formed by pathological dipeptide repeat proteins (DPRs), which is absent in other ALS models (Ramos-Campoy et al. 2018; Mead et al. 2023). Although the implications of these differences for replicating the disease phenotype and mechanisms of neurodegeneration in ALS remain unclear, their unsought effects on the stability and survival of motor units cannot be ruled out.

The discrepancies in the histopathological profiles of preclinical models suggest significant methodological limitations and call for cross-model validation of MN vulnerability data, which have been mainly obtained in SOD1 studies (Baczyk et al. 2022b; Nijssen et al. 2017). They also indicate potential flaws in the therapeutic strategies relying on preclinical data, with a pervasive impact on clinical translation. With over 60 compounds currently in clinical trials and many more tested over the recent two decades, progress in developing disease-modifying therapies has been frustratingly slow, with only three drugs of limited efficacy authorized for use (Mead et al. 2023). Nevertheless, it is reassuring that the approved drugs target pathological mechanisms inferred from preclinical studies. Indeed, the first and the most widely utilised anti-ALS drug, riluzole, inhibits voltage-gated Na^+^ channels and suppresses the presynaptic release of glutamate (Bensimon et al. 1994). A decrease in glutamatergic drive in MNs reinstates the E/I balance in hyperexcitable networks, restores Ca^2+^ homeostasis, and alleviates the ER stress (Bellingham 2011). The second pharmacotherapy approved for ALS, edaravone, augments mitochondrial functions with antioxidant effects, slowing down MN failure and countering the disability progression (Witzel et al. 2022). Finally, the most recently FDA-approved therapy, AMX0035, is a proprietary oral combination of two drugs already in use, sodium phenylbutyrate and tauroursodeoxycholic acid, with beneficial effects attributed to suppression of mitochondria-mediated apoptosis and mitigation of ER stress (Mitsumoto et al. 2022; Paganoni et al. 2020). The intersection of the action mechanisms of anti-ALS therapies with those inferred in MN degeneration by preclinical studies warrant systematic analysis and cross-model validation of the effects of anti-ALS therapies on the vulnerability of motor units in multiple models. Likewise, it would be interesting to investigate the response of different motor unit types to therapeutic leads currently in phase II/III trials with antioxidant (RT001, AP-101), anti-amyloidogenic (ION-363, Tofersen) and anti-inflammatory (Ibadilast, BLZ-945) effects.

## Summary and future directions

**Fig. 3:**
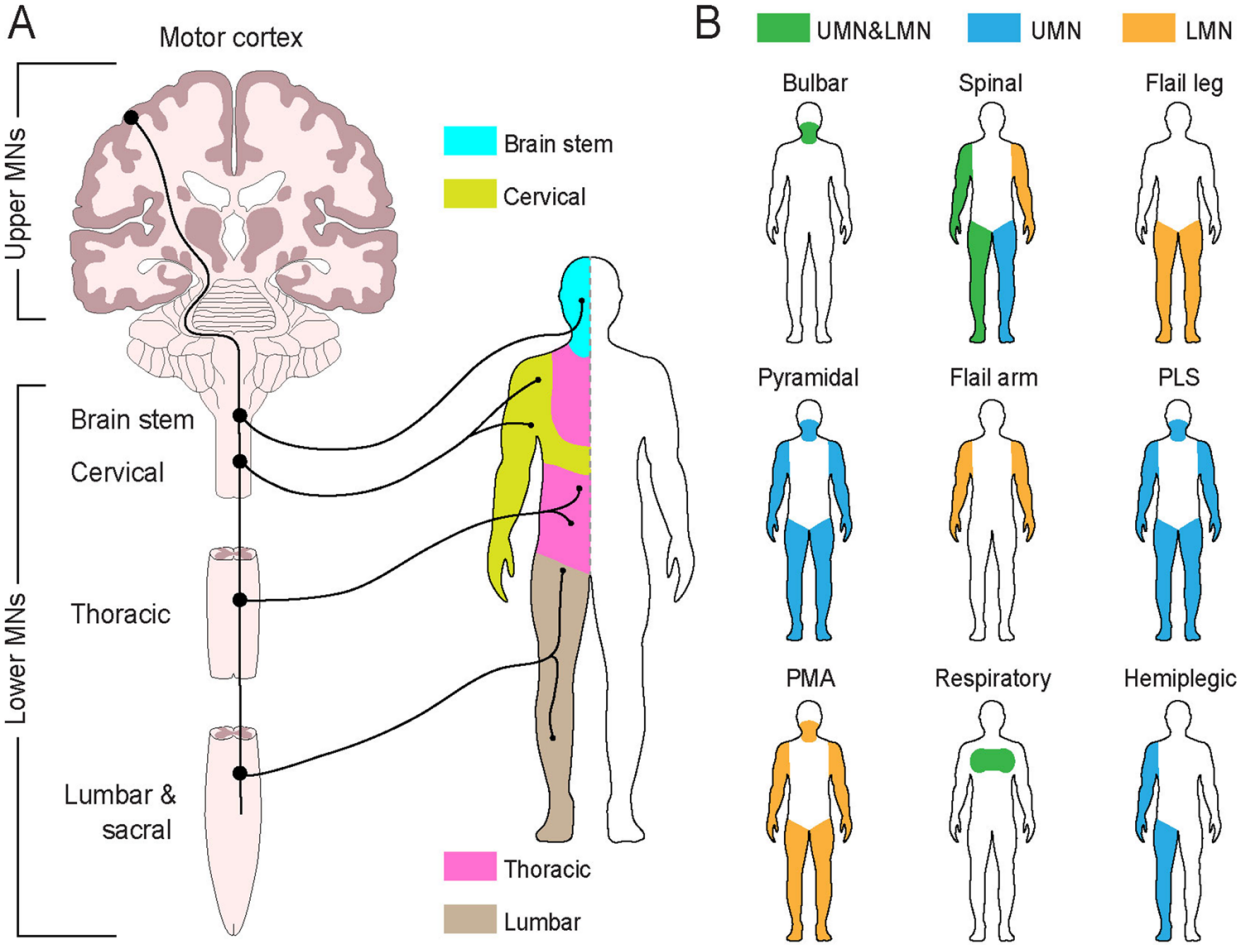
Anatomical perspective on phenotypic variants of ALS with effects on the pattern of motor function involvement. **A** Schematised representation of the human central nervous system with location of distinct functional groups of motor neurons (upper MNs and lower motor neurons, which include brain stem or bulbar, cervical, thoracic, lumbar and sacral) and topographic map of their innervation distribution along the body axis (left and right, respectively). **B** Patten of motor involvement in different ALS phenotypes. *UMN* upper motor neurons, *LMN* lower motor neurons. Depending on the site of the onset of degeneration and structures involved, clinical phenotypes vary from paralysis of a specific group of muscles innervating cranial nerves (bulbar) to some or all limbs (spinal), specific muscles innervating legs (flail leg), all listed areas (pyramidal), arms only (flail arms), all specified above muscles (in primary lateral sclerosis, PLS and primary muscular atrophy, PMA) as well as rare respiratory forms of ALS associated with selective degeneration of MNs innervating the diaphragm and intercostal muscles. Modified from Swinnen and Robberecht (2014) with permission

ALS is the most prevalent degenerative disease of MNs, with no existing cure or treatment to halt neuronal loss or reverse the decline of motor functions available. Although the breakdown of motor units and associated functional decline represent principal hallmarks of the disease and the prime cause of mortality, there is considerable variability in clinical phenotypes attributed primarily to the anatomical location, extent and functions of affected MNs **(**Fig. 3**)**. Notably, the clinical phenotypes of ALS may extend beyond impairements of voluntary motor activities, involving frontotemporal, extrapyramidal and cerebellar impairments, as well as autonomic dysfunctions.

Throughout this article, we revisited molecular and cellular mechanisms implicated in the differential vulnerability of MNs to ALS and discussed factors contributing to the extent of motor decline with neurodegeneration. Analysis of the sensitivity of motor units, in addition to cell-autonomous effects, has uncovered various extrinsic neuronal and non-neuronal factors, supporting the composite nature of the disease. The complex interplay of these effects with genetic, regulatory and environmental factors seems to shape the multifaceted phenotypes of ALS and determine the  trajectory of its progression and prognosis. It is important to note that, like other neurodegenerative proteinopathies, ALS shows prion-like propagation of misfolded TDP-43, SOD1 and FUS proteins throughout the CNS, driving the feed-forward cascade effects and evolution of clinical symptoms. These features, along with numerous factors shaping the differential vulnerability of MNs, may underlie the multisystem features of ALS with a broad spectrum of clinical phenotypes.

Notwithstanding significant mechanistic advances in the pathobiology of ALS, the progress in developing disease-modifying pharmacotherapies has been very slow. The recent arrival of precision therapeutic tools and gene editing technologies inspires optimism for translational breakthroughs in the foreseeable future through genetic reprogramming, transcriptome interference and cell therapy to pave the way for the protection of MNs and rescue their functions (Amado and Davidson 2021; Ovsepian and Waxman 2023). Together with improved animal models and patient-derived tissue studies, these advances should not only enhance our understanding of the limits of MNs, but also expose their reserves to overcome the challenges imposed by ALS.

## Data Availability

This article contains no primary data. All illustrative material presented in this study is original and can be used per the Open Access Policy and Publisher’s guidelines.
